# Physiological Mechanisms behind Differences in Pod Shattering Resistance in Rapeseed (*Brassica napus* L.) Varieties

**DOI:** 10.1371/journal.pone.0157341

**Published:** 2016-06-14

**Authors:** Jie Kuai, Yingying Sun, Tingting Liu, Peipei Zhang, Min Zhou, Jiangsheng Wu, Guangsheng Zhou

**Affiliations:** College of Plant Science and Technology, Huazhong Agricultural University, Wuhan, Hubei Province, P.R. China; Huazhong university of Science and Technology, CHINA

## Abstract

Pod shattering resistance index (SRI) is a key factor affecting the mechanical harvesting of rapeseed. Research on the differences in pod shattering resistance levels of various rapeseed varieties can provide a theoretical basis for varietal breeding and application in mechanical harvesting. The indicators on pod shattering resistance including pod morphology and wall components were evaluated on eight hybrids and open pollinators, respectively, during 2012–2014. The results showed the following: (1) From the current study, SRI varied greatly with variety, and conventional varieties had stronger resistance than hybrid according to the physiological indexes. and (2) Under the experimental conditions, the SRI was linearly related to pod wall weight and the water content in pod walls, and the goodness-of-fit measurements for the regression model of the SRI based on pod wall weight and water content were 0.584** and 0.377*, respectively, reaching the significant level. This illustrated that pod wall weight and the water content in pod walls determined the SRI. (3) Compared with the relative contents of biochemical components in pod walls, the contents of particular biochemical components in pod walls had closer correlations with SRI. Among the biochemical components, the hemicellulose content was the decisive factor for the SRI.

## Introduction

Rapeseed (*Brassica napus* L.) is an important oil crop in China, accounting for ~30% of the world’s planting area and total output [1]. However, the self-sufficiency rate for edible vegetable oil in China is still less than 40% [2]. Mechanized production reduces labor, improves the efficiency of rapeseed production and could improve rapeseed productivity, thereby, ensuring China's edible oil security. Mechanical seeding technologies for rapeseed in China have progressed rapidly, but the proportion of the crop that is mechanically harvested is still relatively low. This is mainly because of a lack of uniform maturity and the susceptibility to pod shattering, resulting in only a short period suited for mechanical operation and a high loss ratio, respectively [3]. Research shows that the suitable operation period, having a loss ratio of less than 8% for mechanical harvesting, is only 3–4 d [4].

Pod shattering resistance in rapeseed is a critical factor affecting mechanized harvesting. Enhancing pod shattering resistance not only lengthens the suitable mechanical operation period, but also decreases the losses from pod shattering, contributing to the extension of mechanized harvesting technologies in rapeseed. Pod shattering resistance is closely correlated with its own traits. Previous research related to pod shattering resistance has produced varying results. According to Morgan, pod shattering resistance is positively correlated with pod wall weight, but not correlated with pod density, pod length and width, or seed number per pod [5]. In other studies, pod shattering resistance was significantly correlated with pod length [6], pericarp weight [7] and vascular bundle size [8]. In addition, the degrees of pod senescence [9] and of cellulose and lignification in the pod wall are related to pod shattering [10–12].

Genetic characteristics are the major factors affecting pod shattering resistance [13]. Most existing studies have focused on a single type or variety, neglecting differences in pod shattering resistance among varieties. We assumed that differences existed in mechanisms behind pod shattering resistance in rapeseed varieties. Accordingly, we chose conventional and hybrid varieties with significantly different levels of pod shattering resistance as materials, and analyzed the biochemical components in pod walls (e.g. cellulose and lignin) for the first time. Additionally, we examined pod shattering resistance and pod traits in different varieties, aiming to elucidate the physiological basis behind the differences in varietal pod shattering resistance levels that underlie their breeding and application for mechanized harvesting.

## Materials and Methods

### Plant material and experimental design

The experiment was conducted in randomized complete bock design in Huazhong Agricultural University (Wuhan, China) during 2012–2014. Each treatment was performed in three replicate plots, each of which had an area of 20.0 m^2^ (10.0 m long and 2.0 m wide). The previous crop (rice) in the rotation was harvested during the first 10 d of September, and soil indicators were measured before rapeseed seeding (Table 1). We selected two conventional varieties (Huahang 901 and Huashuang 5) bred by Huazhong Agricultural University and 14 [eight hybrid (Huayouza 10, Fengyou 520, Zhongyouza 12, Huayouza 62, Huayouza 9, Zhongnongyou 9, Zhongnongyou 6, Dadi 55) and six conventional (Yangguang 2009, Zhongshuang 9, Zhongshuang 11, Zhongshuang 12, 2012-C1103, 2012-C1107)] registered varieties grown in the middle reaches of the Yangtze River as experimental materials.

**Table 1:** The initial soil status of the field in 2012–2014.

Year	pH	Organic matter (g kg^-1^)	Available nitrogen (mg kg^-1^)	Available phosphorus (mg kg^-1^)	Available potassium (mg kg^-1^)
2012–2013	6.2	34.1	96.3	12.1	112.3
2013–2014	6.4	33.8	101.2	14.4	109.2

Rapeseeds were sown at a density of 4.5 × 10^5^ seeds ha^-1^. Each plot consisted of six rows, and the row and plant spacing was 25×8.9 cm in both years. The seed density was evaluated directly after seedling emergence and adjusted for precise planting density at the five-leaf growth stage for all plots. Pest and disease control were performed according to local management practices; 750 kg ha^-1^ of calcium superphosphate and 135 kg ha^-1^ of potassium chloride were manually applied to the soil as basal fertilizer before seeding. Another 135 kg ha^-1^ of potassium chloride was manually applied to the soil during the bolting stage, and 360 kg ha^-1^ nitrogen from urea was used as the N fertilizer, half of which was manually applied to the soil before seeding (basal fertilizer), 20% during the seedling stage, and 30% during the bolting stage. The field was not irrigated during the growing season.

### Sampling and measurements

After the pods on the raceme turned yellow (at ~45 d after the end of flowering), 20 pods on the middle part of the raceme of 10 rapeseed plants per plot were cut and air-dried in containers using the same specifications. Then, the following indicators were measured. Plants in two rows on each side of the plot were discarded to avoid border effects.

#### Pod agronomic traits

Pod length and width were measured using Vernier calipers. According to Morgan’s [5] random collision method with optimization, 20 pods for each plant and eight steel balls having 14 mm diameters were placed into a cylindrical plastic container, with a 14.8 cm diameter and 7.4 cm length, and were oscillated for 10 min in a shaker at 280 rpm with an amplitude of 24 mm (HQ45Z, Zhongke Scientific Instrument and Technology Development Co. Ltd., Wuhan, China). Shattered pod numbers were recorded every 2 min. The pod shattering resistance index (SRI) was calculated as follows:

$$SRI=1-\sum_{i=1}^{5}x_{i}(6-i)/100,$$

where *X*_*i*_ refers to the shattered pod number at the *i*^th^ time (1 ≤ *i* ≤ 5). Ten plants per plot were measured, and the results were averaged. After measuring the SRI, we determined pod wall weight, 1,000-seed weight, seed diameter and seed numbers per pod for the corresponding samples after the removal of the pod septum. After being air-dried, pod walls were weighed (W_1_), placed into a drying oven (DHG-06-200B) and dried to a constant weight at 80°C (48 h). Then, the pod wall was weighed again (W_2_), and the pod moisture content [%, ((*W*_1_ –*W*_2_)/*W*_1_) × 100%] was calculated.

#### Pod wall component determination

Pod walls from each treatment/control were washed thoroughly with distilled water, cut into small pieces, weighed, placed separately into glass vials containing 10 mL of 80% (v/v) ethanol, and heated at 60°C for 30 min. The extract was then filtered and diluted with 80% (v/v) ethanol up to 20 mL [14]. The soluble sugar concentrations were determined in this extract as described by Giannakoula et al. (2008) [15], following the acid-ninhydrin reagent method and the anthrone method [14], respectively. Cellulose, hemicellulose, lignin, acid-insoluble and acid soluble lignin were determined as described by Wu et al. [16].

### Statistical analysis

The meteorological data were kindly supplied by the National Meteorological Information Center. In the current experiment, analysis of variance (ANOVA) was performed using the R package (R Core Team 2014; www.r-project.org). Genotype was used as fixed factor while year and block were used as random factor. Significant differences in means between the treatments were compared by the protected least significant difference (LSD) procedure at P < 0.05. Regression analysis were performed using the mixed model package R Ime 4 to describe the response of SRI to genotypes. The resulting pod wall weight, 1,000-seed weight and seed diameter were used for a cluster analysis according to the Ward’s incremental sum of squares method with SPSS Statistics 20 software (SPSS Inc., Chicago, IL, USA). Figures were prepared using the Origin 9.0 software program.

## Results

### Meteorological data

Climate data for various years are shown in Table 2. For the whole growing duration of rapeseed, the total hours of sunshine and the accumulative temperature in the 2013–2014 season were greater than those for 2012–2013, while rainfall was lower. The difference in accumulative temperature and sunshine hours for the whole growing duration primarily occurred during the overwintering stage, and for the difference in rainfall, during the overwintering and pod stages.

**Table 2:** Meteorological conditions during the growing seasons of rapeseed in 2012–2014.

Growth stages	Climatic index	2012–2013	2013–2014
Seeding to wintering	Precipitation (mm)	78.3	64.4
	Total sunshine duration (h)	1326	1743
	Accumulated temperature (°C)	18694	21256
Wintering to flowering	Precipitation (mm)	77.4	82.7
	Total sunshine duration (h)	1053.0	878.5
	Accumulated temperature (°C)	5805	5262
End of flowering to pod maturity	Precipitation (mm)	169.5	152.4
	Total sunshine duration (h)	1547	1279
	Accumulated temperature (°C)	12176	11857
The whole growth stage	Precipitation (mm)	885.2	791.9
	Total sunshine duration (h)	11884	13030
	Accumulated temperature (°C)	36680	38380

### Difference in the SRI for rapeseed varieties

The SRI varied significantly with variety, and was significantly higher for 2013–2014 than 2012–2013 (Table 3). The coefficient of variation for the 18 varieties reached 71%, indicating a medium variation level. Among the varieties, ‘Huahang 901’ had the strongest pod shattering resistance with an SRI of 0.45, and ‘Fengyou 520’ was the most susceptible to pod shattering with an SRI of 0.00. The average SRI for the 16 tested varieties was 0.13. The SRI values for the eight conventional varieties averaged 0.22, with a variation coefficient of 55% and the SRI values for the eight hybrid combinations averaged 0.04, with a variation coefficient of 109.0%, indicating a high level of variation. The SRI for 2013–2014 followed a similar trend as that for 2012–2013. The data for the 2 years showed that the conventional rapeseed varieties had significantly stronger pod shattering resistance levels than the hybrid combinations.

**Table 3:** Pod shattering resistance of different cultivars in 2012–2014.

Cultivars	2012–2013	2013–2014
Huahang 901	0.45 a	0.95 a
Zhongshuang 12	0.27 b	0.9 ab
Zhongshuang 9	0.25 b	0.85 ab
Zhongshuang 11	0.22 bc	0.84 ab
2012-C1107	0.21 bc	0.8 bc
Yangguang 2009	0.15cd	0.70 c
2012-C1103	0.12 de	0.59 d
Huashuang 5	0.06 ef	0.29 e
Mean	0.22	0.74
CV%	55	29
Zhongyouza 12	0.14 cd	0.24 e
Zhongnongyou 9	0.09d ef	0.22 ef
Huayouza 10	0.05 ef	0.12 fg
Dadi 55	0.03 f	0.10 g
Huayouza 62	0.03 f	0.06 g
Huayouza 9	0.02 f	0.05 g
Zhongnongyou 6	0.01 f	0.03 g
Fengyou 520	0.00 f	0.02 g
Mean	0.04	0.11
CV%	109	79
Significance of variance		
	F value	F _0.05_
Year (Y)	670.386**	4.001
Cultivar (C)	112.516**	1.836
Y×C	32.375**	1.836

Values followed by different letters within the same column are significantly different according to the least significant difference (LSD) test (P< 0.05); Each data represents the mean of three replications. NS means not significant;

*and **means significant differences at 0.05 and 0.01 probability levels, respectively;

CV, coefficient of variation.

### SRI and pod agronomic traits

**Fig 1:**
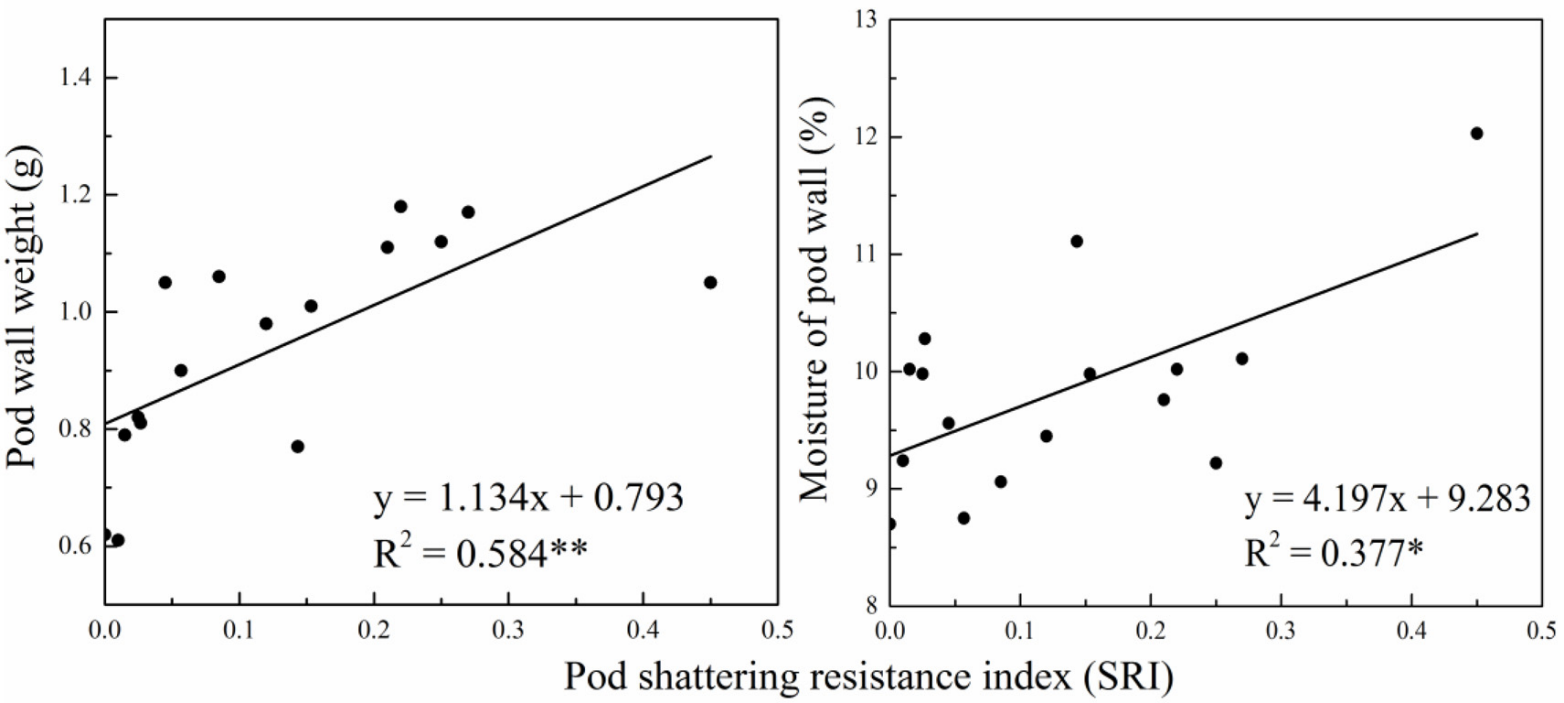
The regression models of SRI with pod wall weight and moisture of pod wall in rapeseed.

Among the various agronomic traits for the tested varieties, pod wall weight, seed weight and pod length had the largest variation (Table 4). The results of the stepwise regression analysis between pod agronomic traits and the SRI further indicated that for these varieties, pod wall weight and the water content in pod walls were the most influential factors on the SRI. Although other indices exerted different levels of influence on the SRI, they were not retained in the regression process. The goodness-of-fit measurements for the regression model (Fig 1) of the SRI based on pod wall weight and water content were 0.584 and 0.377, respectively, reaching the significant level.

**Table 4:** Agronomic characteristics of pods in rapeseed in 2012–2014.

Cultivars	Pod wall weight per 20 pods (g)		1000-seed weight (g)		Seed weight per 20 pods (g)		The seed diameter (×10^−2^ cm)		Pod length (cm)		Pod width (cm)		Beak length (cm)		Seed number per pod		Moisture of pod wall (%)	
	2012–2013	2013–2014	2012–2013	2013–2014	2012–2013	2013–2014	2012–2013	2013–2014	2012–2013	2013–2014	2012–2013	2013–2014	2012–2013	2013–2014	2012–2013	2013–2014	2012–2013	2013–2014
Conventional cultivars																		
Huahang 901	1.05f	1.81c	4.84c	4.12b	2.14d	1.53i	21.3b	18.72c	7.04d	6.27g	0.44h	0.56a	1.45i	1.33n	21.85g	20.78i	12.03a	9.72g
Zhongshuang 12	1.17b	1.92a	4.76d	4.07d	2.10e	1.84c	19.7e	17.89f	6.14i	6.31f	0.56a	0.48e	1.77c	1.56d	21.2h	22.55d	10.11d	9.78f
Zhongshuang 9	1.12c	1.42f	4.70e	3.62g	2.32c	1.55h	21.1c	19.99a	8.15b	7.90a	0.43i	0.43i	1.63e	1.42k	20.25j	21.45g	9.22j	10.03e
Zhongshuang 11	1.18a	1.87b	5.70a	4.34a	3.18a	1.94a	22.0a	18.27e	8.64a	6.47d	0.46f	0.51b	1.64d	1.39m	20.55i	22.37e	10.02e	10.07d
2012-C1107	1.11d	1.41g	4.66f	3.41i	2.04f	1.44l	19.2g	19.87b	6.85f	6.52c	0.52b	0.46g	1.52g	1.63c	20.22j	21.08h	9.76g	10.02e
Yangguang 2009	1.01g	1.45e	4.20h	3.67f	1.95g	1.41m	18.9h	18.49d	5.39m	5.87h	0.50d	0.46g	1.35m	1.87a	22.87f	19.20l	9.98f	9.72g
2012-C1103	0.98h	1.48d	3.98i	3.37jk	1.87j	1.64e	18.8i	17.45h	5.26n	6.42e	0.56a	0.50c	1.39l	1.48g	20.20j	24.42a	9.45i	10.46b
Huashuang 5	0.90i	1.38h	5.52b	3.36k	1.68l	1.92b	20.8d	17.56g	6.29h	6.47d	0.42j	0.50c	1.16n	1.41l	20.17j	24.00b	8.75l	11.00a
The hybrids																		
Zhongyouza 12	0.77f	0.91g	3.14c	3.60c	1.69e	0.98g	18.0b	17.20b	5.39g	5.58e	0.50ab	0.40g	1.41h	1.50d	26.00c	18.93e	9.56e	8.74f
Zhongnongyou 9	1.06a	1.40a	4.58a	3.42d	2.35a	1.49d	19.6a	17.22a	6.94b	7.01a	0.51a	0.46d	1.43g	1.44g	23.93d	17.07h	11.11a	10.02b
Huayouza 10	1.05b	1.41a	2.79f	4.10a	1.01h	1.78a	16.0g	17.22a	7.23a	5.67c	0.51a	0.49a	1.90a	1.33h	17.15g	20.63c	9.06g	10.11a
Dadi 55	0.81d	1.23c	2.99e	3.78b	1.46f	1.56c	17.2e	17.10c	6.34c	5.47f	0.46d	0.46d	1.51e	1.46e	23.32e	23.22a	10.28b	8.99c
Huayouza 62	0.82c	1.11e	3.34b	3.09h	1.93b	1.61b	17.5d	16.66d	5.91e	5.61d	0.48c	0.41f	1.63c	1.56b	27.40b	22.28b	9.98d	8.79e
Huayouza 9	0.79e	1.27b	3.13c	3.21g	1.91c	1.48de	17.8c	16.55e	5.85f	5.82b	0.48c	0.48ab	1.45f	1.77a	29.13a	18.52f	10.02c	8.75f
Zhongnongyou 6	0.61h	1.15d	2.41g	3.36ef	1.71d	1.48de	17.3e	16.23f	5.86f	5.27g	0.48c	0.44e	1.89b	1.52c	23.85d	17.95g	9.24f	8.75f
Fengyou 520	0.62g	1.08f	3.03d	3.38e	1.28g	1.35f	16.3f	17.22a	5.98d	5.01h	0.45e	0.47c	1.54d	1.45ef	20.23f	20.00d	8.70h	8.97d
CV (%)	19.7	20.3	25.6	10.1	25.7	15.2	9.6	6.2	15.0	11.8	8.6	8.3	12.8	9.8	13.9	10.4	8.7	7.3

Values followed by different letters within the same column are significantly different according to the least significant difference (LSD) test (P< 0.05);

Each data represents the mean of three replications. CV, coefficient of variation.

### Classifications of different rapeseed varieties

**Fig 2:**
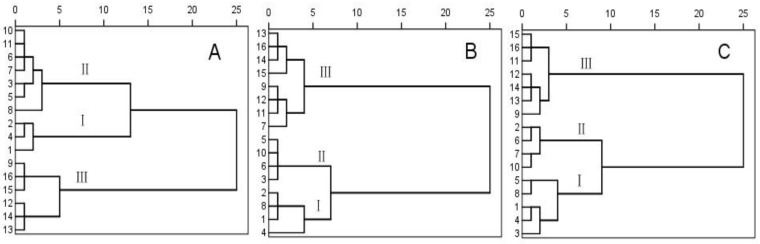
The cluster analysis of rapeseed pod based on pod wall weight (A), 1000-seed weight (B) and the seed diameter (C). Digital 1–16 represent Huahang 901, Zhongshuang 12, Zhongshuang 9, Zhongshuang 11, 2012-C1107, Yangguang 2009, 2012-C1103, Huashuang 5, Zhongyouza 12, Zhongnongyou 9, Hayouza 10, Dadi 55, Huayouza 62, Huayouza 9, Zhongnongyou 6, Fengyou 520, respectively.

Agronomic traits, including pod wall weight, 1,000-seed weight and seed diameter, were highly relevant to the rapeseed SRI (Table 4). A clustering analysis was conducted based on pod wall weight, 1,000-seed weight and seed diameter (Fig 2). Based on the three pod traits and the results of the clustering analysis (Table 5), the 16 varieties were classified into three groups: Group I (‘Huahang 901’, ‘Zhongshuang 12’, ‘Zhongshuang 11’ and ‘Huangshuang 5’), Group II (‘2012-C1107’, ‘Yangguang2009’, ‘2012-C1103’, ‘Huashuang 5’, ‘Huayouza 10’ and ‘Dadi 55’) and Group III (‘Zhongyouza 12’, ‘Zhongnongyou 9’, ‘Huayouza 62’, ‘Huayouza 9’, ‘Zhongnongyou 6’ and ‘Fengyou 520’). Among them, the conventional varieties generally fell into Groups I and II and hybrid combinations into Groups II and III. In Groups I to III, the trends in pod agronomic traits corresponded with those of the SRI.

**Table 5:** Results of the cluster analysis.

Classification	Cultivars
I	Huahang 901, Zhongshuang 12, Zhongshuang 9, Zhongshuang 11
II	2012-C1107, Yangguang 2009, 2012-C1103, Huashuang 5, Huayouza 10, Dadi 55
III	Zhongyouza 12, Zhongnongyou 9, Huayouza 62, Huayouza 9, Zhongnongyou 6, Fengyou 520

### Main components in pod walls and correlations with the SRI

Over the 2 years, the soluble sugar concentration in the different rapeseed groups followed the order of Group I and Group II > Group III, and for hemicellulose concentration, the order was Group I > Group II > Group III. The lignin concentrations for the different categories followed the order Group I > Group II > Group III. (Table 6).

**Table 6:** The biochemical components contents of pod wall and their correlations with pod shattering resistance in 2012–2014 (×10^−2^ g per pod).

Classification	Cultivars	Soluble sugar		Hemicellulose		Cellulose		Acid insoluble lignin		Acid soluble lignin		Total lignin	
		2012–2013	2013–2014	2012–2013	2013–2014	2012–2013	2013–2014	2012–2013	2013–2014	2012–2013	2013–2014	2012–2013	2013–2014
I	Huahang 901	0.15a	0.39b	1.72a	2.91a	1.52a	2.47a	0.52c	1.05b	0.20f	0.19b	0.72c	1.24b
	Zhongshuang12	0.12c	0.42a	1.57b	2.21b	1.31c	2.17c	0.70a	1.31a	0.28a	0.24a	0.98a	1.55a
	Zhongshuang11	0.13b	0.35c	1.63c	2.11c	1.38b	2.32b	0.66b	0.97c	0.23c	0.24a	0.89b	1.21c
	Huashuang 5	0.12c	0.24d	1.27d	1.39d	1.00d	1.71d	0.36d	0.73d	0.19g	0.13c	0.55d	0.86d
	Mean	0.13	0.35	1.55	2.16	1.30	2.17	0.56	1.02	0.23	0.20	0.79	1.22
II	Zhongshuang 9	0.17a	0.27d	1.56b	1.66b	1.41b	1.52e	0.62a	0.86d	0.24a	0.19a	0.86a	1.04d
	2012-C1107	0.13c	0.26e	1.82a	1.54e	1.30c	1.70b	0.57b	0.95c	0.23b	0.16c	0.80b	1.11c
	Yangguang 2009	0.14b	0.30c	1.54bc	1.61c	1.54a	1.69bc	0.43c	0.77e	0.22c	0.18b	0.66c	0.95e
	2012-C1103	0.12d	0.31b	1.37d	1.60cd	1.25d	1.89a	0.41d	0.99b	0.21d	0.19a	0.62d	1.17b
	Zhongnongyou 9	0.11e	0.33a	1.29e	1.89a	0.71e	1.59d	0.40e	1.07a	0.12e	0.14d	0.52e	1.21a
	Mean	0.13	0.29	1.52	1.66	1.24	1.68	0.49	0.93	0.20	0.17	0.69	1.10
III	Zhongyouza 12	0.09c	0.20e	1.32a	0.98e	0.95a	1.24d	0.46b	0.49f	0.15a	0.12b	0.61a	0.61g
	Huayouza 10	0.10b	0.26a	1.10b	1.79a	0.62c	1.57a	0.48a	1.10a	0.08c	0.13a	0.56c	1.23a
	Dadi 55	0.08d	0.26a	1.00d	1.27b	0.56d	1.33c	0.44d	0.74c	0.10b	0.10d	0.54e	0.84d
	Huayouza 62	0.09c	0.24b	0.95e	1.03d	0.74b	1.21e	0.45c	0.75b	0.10b	0.12b	0.55d	0.87c
	Huayouza 9	0.11a	0.26a	1.03c	1.25c	0.38g	1.13f	0.48a	0.75b	0.10b	0.13a	0.58b	0.88b
	Zhongnongyou 6	0.07e	0.22d	0.72f	1.04d	0.51e	0.81g	0.24f	0.59e	0.07d	0.12b	0.30g	0.71f
	Fengyou 520	0.08d	0.23c	0.63g	0.87f	0.45f	1.54b	0.32e	0.68d	0.08c	0.11c	0.40f	0.79e
	Mean	0.09	0.24	0.96	1.18	0.60	1.26	0.41	0.73	0.10	0.12	0.51	0.85
CV (%)		24.8	21.6	27.5	33.9	42.0	27.2	25.9	24.7	42.8	28.7	28.2	24.0

Values followed by different letters within the same column are significantly different according to the least significant difference (LSD) test (P< 0.05);

Each data represents the mean of three replications; CV, coefficient of variation.

**Fig 3:**
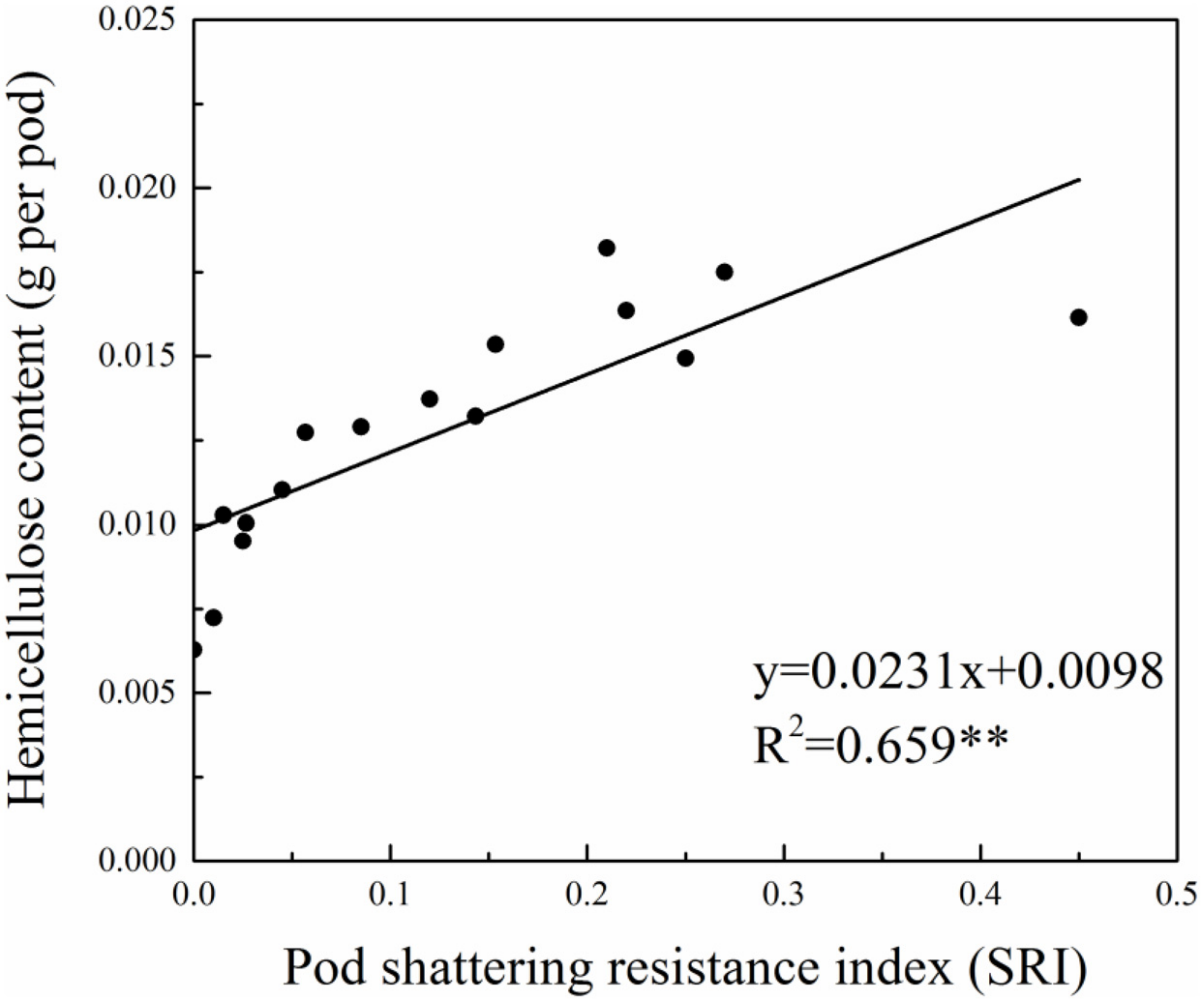
The regression model of pod shattering resistance with the hemicellulose content per pod in rapeseed.

The stepwise regression analysis between physiological indicators and the SRI indicated that for tested varieties, the total amount of hemicellulose per pod wall was the most influential factor on the SRI, and was retained in the regression equation *y* = 0.0231*x* + 0.0098 (where *y* indicates the hemicellulose content in the pod wall, and *x* is the SRI) (Fig 3). The goodness-of-fit measurement for the regression model for the total amount of hemicellulose and the SRI was 0.659, reaching the significant level.

## Discussion

Pod shatter can occur both prior to harvest due to adverse weather conditions and at harvest due to impact from combine harvesters [17]. In the experiment, the SRI for the 2013–2014 season was significantly higher than for the 2012–2013 season, indicating that climatic factors significantly affected pod growth and the formation of pod agronomic traits in rapeseed, which further affected the SRI. This was in accordance with the results of previous research [17]. For the whole growing duration, the total hours of sunshine and the accumulative temperature for 2013–2014 were greater than for 2012–2013, while the rainfall level was lower. The difference in the accumulative temperature and sunshine hours during the growing season primarily occurred during the overwintering stage. Relatively long sunshine hours and high accumulative temperatures were beneficial to rapeseed growth and development during the seedling stage, as well as to carbohydrate accumulation, which laid the material foundation for the development of pods. The difference in rainfall between the 2 years primarily occurred in the overwintering and pod stages. According to Grant’s (1996) research on the genus Lotus (*Fabaceae*), the humidity during the maturity stage is an important factor affecting silique shattering and the critical relative humidity level for different genotypes is within the range of 35%–49% [18]. In the present study, rainfall during the maturity stage in the 2012–2013 season was higher than in the 2013–2014 season, causing higher relative humidity, which likely aggravated pod shattering. Therefore, relatively long sunshine hours and high accumulative temperatures during the overwintering period, along with less rainfall during the pod stage, enhanced rapeseed pod shattering resistance.

In addition to environmental factors, rapeseed pod shattering resistance is greatly affected by genetic factors [19–21] and varies with variety [22]. Given that previous research results on pod shattering resistance and agronomic traits in rapeseed were not fully consistent under various experimental conditions, we measured the SRI for 16 rapeseed varieties (eight conventional and eight hybrid) using the optimized random collision method in 2012–2014 to further explore the relationship between pod agronomic traits and the SRI. The results showed that the SRI significantly varied with variety and that, among the tested rapeseed material, the conventional varieties had significantly stronger pod shattering resistance values and less intervarietal variation compared with hybrid combinations. Therefore, compared with hybrid rapeseed, conventional rapeseed is more likely to provide stable varieties or breeding materials. The correlation analysis illustrated that the SRI was significantly and positively correlated with pod wall weight and seed diameter; and significantly positively correlated with pod length and 1,000-seed weight, in accordance with the results of Child [8] and partly contradictory to those of Morgan [5]. Rapeseed pod shattering resistance is closely related to its water content [23]. In the present study, the SRI and water content in air-dried pod walls were positively correlated. On the one hand, the high water content and hydrotropic contents, including protein, starch and cellulose in pod walls, were beneficial to the growth, development and structural stability of pods. On the other hand, the moisture affected metabolism inside the pods [24] and delayed pod aging, thus reducing pod shattering during the suitable harvesting period. We further explored the relationships between pod agronomic traits and the SRI. A stepwise regression analysis showed that the SRI was linearly related to pod wall weight and the water content in pod walls, and these were retained in equations following significance testing. This illustrated that among the agronomic traits, pod wall weight and the water content in pod walls could be used as important indicators for screening elite rapeseed resources having pod shattering resistance. Rapeseed pod wall weight and the water content in pod walls were closely correlated with the level of hydrotropic substances in pod walls. However, to the best of our knowledge, there are no reports on intervarietal differences in the contents and composition of these hydrotropic substances and the mechanisms behind their influence on pod shattering resistance. Thus, further research is required.

Rapeseed pod shattering is a complex physical, physiological and biochemical process. Upon reaching physiological maturity, pods will shatter when the external force is greater than the connective force between pod pericarps. The dry weight of the pod wall reflects its plumpness, and the SRI is most closely correlated with pod wall weight. Physiologically, pod shattering resistance is essentially related to the amount and composition of carbohydrates in the pods. Carbohydrates, such as cellulose and lignin, are important constituents of the pod cell walls, which directly affect pod mechanical shattering resistance. Cellulose ensures the toughness of cell walls, while lignin, an important phenolic compound with a complex structure in plants, enhances the hydrophobicity and hardness of cell walls, physical strength and water conducting capacity, and other important functions in the plant body [25]. Previous studies mainly focused on the relationship between the contents of lignin and cellulose, and stem lodging resistance in other crops [26–28]. A lignin deficiency in stems aggravates lodging in rice [26] and wheat [22]. Wheat varieties susceptible to lodging have lower lignin and hemicellulose contents, and lower accumulation levels in stems compared with those resistant to lodging [28]. However, rapeseed pod shattering resistance and the carbohydrate contents in pod walls have not previously been reported. A study on other crops indicated that high cellulose (and hemicellulose) and lignin contents in chickpea pods impede the separation of cells in the separation layers and reduce pod damage [29]. After pod dehydration, due to varying levels of shrinkage, endogenous tension generates between lignified and non-lignified cells. At the same time, cells in the separation layers detach from each other under the action of hydrolases. The interaction of the two events leads to pod shattering [30]. Differences in lignin or cellulose contents in pods of different rapeseed varieties can change the structure of the separation layer, and thus affect pod shattering. The results of factor analyses revealed that the hemicellulose, cellulose and lignin contents in the pod walls made the greatest contributions to rapeseed SRI. Thus, a higher total amount of cellulose or hemicellulose enhanced the cell’s mechanical support in the separation layer, as well as pod shattering resistance in rapeseed. The increased lignin content in rapeseed pods improves the lignification level at the carpel connections [31] and thickens cell walls in the separation zone. Additionally, microtubules that connect carpels through fiber bundles in the carpopodium are closer to the inside edges of the zone [10]. These changes help reduce pod shattering and enhance pod shattering resistance. According to the stepwise regression test, the SRIs for different varieties were linearly related to the amount of total hemicellulose in pod walls, which was retained in equations following significance testing. This illustrated that pod wall weight affected pod shattering resistance by regulating the total hemicellulose amount in the pod walls.

## Conclusions

We determined that the conventional rapeseed varieties had significantly higher SRIs, indicating that they will provide more stable varieties or breeding materials having stronger pod shattering resistance. The rapeseed SRI was most closely related to pod wall weight, but the biochemical components in pod walls were the determining factor. Among the biochemical components, the hemicellulose content was the decisive factor in the SRI, generating the equation *y* = 0.0231*x* + 0.0098 (where *y* indicates the hemicellulose content in the pod wall and *x* is the SRI). The relationships between pod morphological indicators and the SRI varied under different experimental conditions, but the hemicellulose content was a stable physiological indicator affecting pod shattering resistance. Therefore, in addition to observations of rapeseed pod morphology, we should screen elite resources for pod shattering resistance, and improve pod shattering resistance by combining pod wall components.

## Supporting Information

S1 Dataset This excel file includes pod agronomic traits, pod wall biochemical component in rapeseed varieties. The tables and figures in the paper are based on this dataset.
(XLSX)
